# Development of a Monte Carlo model for treatment planning dose verification of the Leksell Gamma Knife Perfexion radiosurgery system

**DOI:** 10.1120/jacmp.v17i4.6196

**Published:** 2016-07-08

**Authors:** Jiankui Yuan, Simon S. Lo, Yiran Zheng, Jason W. Sohn, Andrew E. Sloan, Rodney Ellis, Mitchell Machtay, Barry Wessels

**Affiliations:** ^1^ Case Medical Center, University Hospitals of Cleveland Cleveland OH USA

**Keywords:** Gamma Knife, radiosurgery, Monte Carlo, source model

## Abstract

Detailed Monte Carlo (MC) modeling of the Leksell Gamma Knife (GK) Perfexion (PFX) collimator system is the only accurate *ab initio* approach appearing in the literature. As a different approach, in this work, we present a MC model based on film measurement. By adjusting the model parameters and fine‐tuning the derived fluence map for each individual source to match the manufacturer's ring output factors, we created a reasonable virtual source model for MC simulations to verify treatment planning dose for the GK PFX radiosurgery system. The MC simulation model was commissioned by simple single shots. Dose profiles and both ring and collimator output factors were compared with the treatment planning system (TPS). Good agreement was achieved for dose profiles especially for the region of plateau (<2%), while larger difference (<5%) came from the penumbra region. The maximum difference of the calculated output factor was within 0.7%. The model was further validated by a clinical test case. Good agreement was obtained. The DVHs for brainstem and the skull were almost identical and, for the target, the volume covered by the prescription (12.5 Gy to 50% isodose line) was 95.6% from MC calculation versus 100% from the TPS.

PACS number(s): 87.55.dk

## I. INTRODUCTION

The Leksell Gamma Knife (LGK) has been used extensively in the standard of care for the treatment of patients with brain tumors, arteriovenous malformations, and functional disorders.^(1)^ It provides a noninvasive alternative for patients when traditional brain surgery is not an option. The new model Perfexion (PFX) of the LGK system, introduced in 2006, has a completely different collimator system with respect to its predecessors. The PFX uses 192 Cobalt‐60 sources which are organized into eight independent position‐controlled movable sectors of 24 sources each. The stationary and built‐in portion of the collimator system has three apertures for each source — 4, 8, and 16 mm. In order to change the field size, each sector can be moved to the corresponding collimator set by servo‐controlled motors located at the rear of the radiation unit. It thus eliminates the need for labor‐extensive manual installation of the collimator helmets as in the older models. With an expanded treatment area and enhanced accuracy, the new system allows clinicians to treat tumors that were unreachable with previous technology.

The Leksell GammaPlan (LGP) is the computer‐based treatment planning system (TPS) that is specifically designed to plan the dose delivered by the device. Prior to LGP version 10, the dose algorithm used in the system is so‐called TMR classic, which assumes the whole head has

a homogenous density as water. This assumption leads to errors in the calculation of radiation dose near heterogeneities such as bone and air cavities. From version 10, the LGP planning system provides an improved TMR classic algorithm, named TMR 10,^(2)^ with updated fitted parameters from more accurate Monte Carlo (MC) simulations and measurements. In TMR 10 algorithm, the whole head as water is still assumed. The other dose algorithm provided in the LGP is the CT‐based convolution algorithm.^(3)^ An additional CT scan is required for this algorithm to obtain the electron density information. The CT image provides more reliable skull‐shape definition with computer skin surface rendering, and it allows the convolution dose algorithm to calculate more accurately for the dose scattering and the inhomogeneity correction. Recently study has shown that the convolution algorithm can yield dose difference up to 11.5% compared with the TMR classic dose algorithm.^(4)^

The MC simulation has proven to be the most accurate dose algorithm, especially for small irradiating beams and wherever heterogeneity exists as presented in the LGK. In radiosurgery usually with a single high‐dose fraction, lacking or improperly accounting for inhomogeneity may have profound clinical implications in terms of tumor control and complications. It is important that the electronic inequilibrium and secondary scattering are taken into account properly. Studies on MC simulations are mainly for the prior LGK systems because the collimator system for prior LGK models is relatively simpler to implement.^5^, ^6^, ^7^, ^8^, ^9^ To simulate prior LGK, only one dataset of the phase space of photon beams at the distal source channel is needed since this phase space is identical for all 201 sources. For LGK PFX, however, only a handful of MC studies are found in the literature because the Monte Carlo simulation for the PFX model is more complex due to the noncoaxial source arrangement and complicate modeling of the collimator system.^10^, ^11^ Existing general purpose MC packages have been employed for these studies. For example, Battistoni et al.^(12)^ used the FLUKA code^(13)^ to simulate the PFX for homogenous water phantom. Best^(14)^ reported a MC model of one sector of PFX with detailed source modeling based on the PENELOPE MC code,^(15)^ and most recently, Pipek et al.^(16)^ presented a MC model based on the Geant4 package.^(17)^ Detailed source modeling, however, requires the internal geometrical description of the collimator system and source arrangement including material data. To acquire this information, nondisclosure agreement must be signed with Elekta Instrument AB, as stated in the above studies.

In this work, we present an approach without using the detailed geometrical information of the PFX collimator system. Instead, we created a virtual source model based on film measurement. As the main purpose of our work is to develop a patient‐specific dose verification tool, similar to the study by Mamalui‐Hunter et al.,^(18)^ for LGK PFX, we postulate that the *ab initio* source model is not critical as long as the calculated dose profile for each collimator from the virtual source model agrees with the LGP‐calculated dose profile. This technique is similar to the method used in the commissioning process for conventional linacs, where a source model is first presumed and then validated in water phantom by evaluating the calculated percentage depth dose and profiles.

## II. MATERIALS AND METHODS

### A. Film measurement to derive fluence distribution

In order to calculate dose delivered to the phantom, fluence distribution from each individual source is required. Preferably, this information is obtained from detailed Monte Carlo modeling of the collimator system based on geometrical description and source arrangement, as noted in various studies.^10^, ^11^, ^12^, ^13^, ^14^, ^15^, ^16^ In our work, however, we derived fluence distribution from film measurement for the reasons that 1) the internal geometrical information of the collimator system is not available to us, and/or fully simulations of the individual source and collimator is out of our capability due to the great complexity; and 2) the technique that uses film measurement to derive fluence distribution has been successfully employed in dose delivery verification for conventional and intensity‐modulated radiation therapy (IMRT).^(19)^ It is interesting to investigate if this technique can be applied to LGK PFX Monte Carlo simulations for the purpose of developing a dose verification tool.

To measure fluence distribution for each individual source, we adopted the panoramic imaging method, similar to the work by Cho and colleagues.^(20)^ The main purpose of their work was to verify the source and collimator configurations of PFX to check the mechanical integrity against manufacture specification. Our goal, instead, is to build a virtual source model to perform patient‐specific independent quality assurance (QA) for PFX based on the derived fluence of individual source.

**Figure 1:**
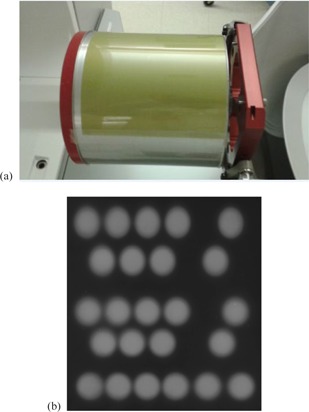
A cylindrical phantom (a) installed in the Leksell Gamma Knife frame for irradiation; 5 mm bolus on the top of the EBT film is not shown in the figure. Source images (b) of 16 mm collimator for one sector on the film.

The panoramic method uses radiochromic films (EBT2, International Specialty Products, Waynes, NJ) to wrap around a cylinder phantom. Observing that the overlap of source images on the film for the 16 mm collimator was still substantial even using the cylinder phantom with a diameter of 152 mm in Cho et al.'s work,^(20)^ we created a larger cylinder phantom with a diameter of 226 mm (created by University Hospital's workshop) so that each individual source on the film for all three collimator sizes were well separated, as shown in Fig. 1(a). The isometrics of the cylinder was estimated to be less than 1 mm by measuring the diameter of the cylinder. 5 mm bolus was put on the cylinder as the buildup. To avoid the overlap from other directions, only the first sector was used to irradiate the films for about 30 min. The films were then scanned with Epson Flatbed Scanner (Expression 11000XL model, Epson America Inc., Long Beach, CA) with the resolution of 240 dpi, 150 dpi, and 96 dpi for collimator 4 mm, 8 mm, and 16 mm, respectively. A clinical film dose calibration curve generated with Linac 6 MV up to 20 Gy was applied, since EBT2 film has been shown to have very low‐energy dependence for X‐ray beam irradiations.^(21)^

The film analysis was carried out using an in‐house developed MATLAB program (MathWorks, Natick, MA). The characterization of each individual source was extracted by the program to avoid subjective interpretation error. An illustration of irradiated film for 16 mm collimator is shown in Fig. 1(b). To separate individual source, the source images from each sector were first smoothed using the anisotropic diffusion method,^(22)^ and then the morphological operation(^23^) was applied, which is an erosion followed by a dilation with a structure element of the disk shape. The dosimetric center of each source was determined with the weight of the pixel intensity. With the coordinates of the center of each source and known cylinder radius, we were able to figure out the latitudinal and azimuth angles of each source in a sector. As a sanity check, we compared the results with the manufacturer's data. We found that the difference of latitudinal location for all shots at five rings was less than 0.14 mm, and the difference of the azimuth angles between the measurement and the manufacturer data was less than 0.2° for the three collimators. This good agreement indicates the correctness of overall measurement setup. In the MC simulation, however, we still employ the published manufacturer data^(19)^ for the source latitudinal and azimuth angles instead of our measured. The sector fluence includes 24 individual fluence maps of each source, and the full fluence of a shot (a shot is a configuration of 8 sectors) consists of 8 sector fluences by rotating the sector by every 45°.

### B. Virtual source model and Monte Carlo simulation

Our virtual source model for the PFX MC simulations consists of photon energy spectrum, approximated single point source and derived fluence distribution of each individual source in one sector. For energy spectrum, we used published data from Fig. 13 in the work by Best^(14)^ The spectrum was generated by detailed MC simulation of the PFX collimator system with elaborated geometry information using Penelope MC code. The two energy peaks for C60o (1.17 MeV and 1.33 MeV) are present along with two apparent Compton scattering edges (1.12 MeV and 0.96 MeV). The same normalized spectrum was used for three collimators. To model the source precisely, the simulation should include 1) the description of source pellet configuration, which is a linear source having 2 cm in length; 2)taking into account of the source bushing as the primary collimator; 3) the geometrical description of concentric cylinders of various diameters in the 4, 8, and 16 mm collimator; and 4) the alignment of the source and the collimator which is coaxial for the 4 mm collimator, but it is not the case for 8 and 16 mm collimators as a small angle deviation exists for these two collimators. For this work and for the reasons mentioned above, we made a simple approximation. We assumed that the shape of an individual source is a single point and the line connecting the source point and the focus point passes through the center of the 2D fluence distribution, as described above. The distance from the source point to the focus point is different for each of the five rows of sources within a sector. The coordinates in the cylindrical system represented by the azimuth direction θ and latitudinal direction φ of the sources in a sector were taken from Table 1 in the work by Cho et al (20) In the MC simulation, a source particle was sampled for the derivation fluence distribution. The planned shot time was assigned to its weight. Since the scanned data is in Cartesian 2D geometry, we need to convert the sampled location to the cylindrical coordinate system (x, y, z) of the phantom as follows:

(1) $$x=R\cdot \text{cos}(x_{S}/R),y=R\cdot \text{sin}(x_{s}/R),z=y_{s}$$

where *R* is the radius of the cylindrical phantom, and $x_{s}$ and $y_{s}$ are the sampled locations from the fluence distribution at the film plane. The direction of the particle was determined as the vector connecting the source point with the sampled initial position.

The transport of the particles was carried out using our in‐house developed MC code, which has been described in our previous work.^(24)^ Briefly, the three physics processes (i.e., Compton scattering, photoelectric ionization, and pair production) were considered in the code for photon transport, and the class II condensed history method was used for electron transport. The hard interactions, such as inelastic collision and bremsstrahlung, were simulated explicitly for energies above certain cutoffs. The continuous slowing down approximation (CSDA) was employed for energies below the thresholds. The cutoff energy for absorption was set to 50 keV for photons and 200 keV for electrons in our simulations, which is corresponding approximately to a mean free path of 1 mm (the voxel size in our calculations was set to 1 mm). The simulation statistical uncertainty in our work is defined as the maximum uncertainty for the voxels of the region of 50% of the maximum dose.^(25)^ Message Passing Interface (MPI) was implemented in the code to take advantage of multiprocessor computing resource. The MC code is well validated and has been successfully applied in TomoTherapy MC dose calculations.^(26)^

**Table 1:** Ring and collimator output factors

*Collimator (mm)*	*Ring*	*Calculated*	*Manufacturer Data*	*Comparison*
4	1	0.811	0.812	−0.1%
	2	0.826	0.823	+0.4%
	3	0.791	0.795	−0.5%
	4	0.731	0.726	+0.7%
	5	0.664	0.664	0.0%
	Total	0.814	0.814	0.0%
	Calc	0.809	0.814	−0.6%
8	1	0.939	0.934	+0.5%
	2	0.920	0.919	+0.1%
	3	0.875	0.874	+0.1%
	4	0.784	0.782	+0.3%
	5	0.706	0.708	−0.3%
	Total	0.901	0.900	+0.1%
	Calc	0.894	0.900	−0.7%
16	1	0.961	0.961	0.0%
	2	1.000	1.000	0.0%
	3	0.980	0.981	−0.1%
	4	0.917	0.914	+0.3%
	5	0.849	0.847	+0.2%

### C. Output factors and MC dose calibration

The ring output factor is defined as the ratio of the dose rate from the sources at the same ring to the dose rate from the sources at the second ring of the 16 mm collimator which has the highest dose rate, that is

(2) $$f_{cr}=D_{cr}/D_{16,2}$$

where $f_{\text{cr}}$ is the ring output factor for collimator c and the r‐th ring, and $D_{16,2}$ is the dose rate from the sources at the second ring of the 16 mm collimator. These values cannot be directly measured as there is no mechanism to open the sources at the same ring alone. Monte Carlo simulation is the only approach to determine these values. The collimator output factor $f_{c}$ is summation of the ring output factors over five rings for each collimator, weighted by the number of sources at the ring. The collimator output factor can be measured experimentally.

Full detailed MC source modeling is capable to calculate the ring output factor as the geometrical and material information is known to the program. The film measurement based virtual source model in this work, however, cannot be used directly to determine accurate output factors for the reasons that: 1) the measured data is not the fluence but the dose after the dose calibration curved is applied; 2) the buildup of the film measurement will distort the measured result; and 3) the misalignment of the source axis and the collimator does not take into account.

Therefore, for this work, rather than attempted to carry out an *ab initio* output factor calculations, we adjusted the parameters such as the source‐to‐focus distance^(27)^ to match the manufacturer‐provided ring output factors. The source‐to‐focus distances for each ring provided in the work by Petti^(27)^ were determined by fitting a beam model to the manufacturer MC generated data. As long as the ring output factors are correct, the collimator output factors are correct as well.

In order to obtain absolute dose, a calibration factor is needed to convert MC data which is in the unit of dose per simulated particle to the dose in the unit of Gy. Specifically, we performed the MC simulation with a spherical phantom of 8 cm radius using one shot of 16 mm collimators and compared the dose distribution to the LGP TPS‐calculated dose with the same phantom setting. The calibration factor M which converts the MC result in unit of Gy/particle to the dose Gy is defined as

(3) $$D_{m}=M/\Omega \times D_{r}\times T\times D_{MC}$$

where $D_{m}$ is the central point dose from LGP TPS; $D_{\text{MC}}$ is the MC result at the same point; $D_{r}$ is the treatment dose rate, and *T* is the treatment time. The prescription was set to 5 Gy to 50% isodose line. *Ō* is the total solid angle, which can be calculated as

(4) $$\Omega =\sum [S_{S}/(R_{S}-L/sin\theta_{s})2]$$

where $S_{s}$ is the summation of the pixel area of the fluence map for the source s, $R_{s}$ is the source‐to‐focus distance, *L* is the radius of the cylinder phantom, and $\theta_{s}$ is the latitudinal angle of the source. To match the manufacturer's ring output factors, two parameters were adjusted, that is the source‐to‐focus distance and the threshold of the mask filter for fine‐tuning the source image. Compared with the parameters provided in the work by Petti,^(27)^ the maximum differences of our fitted source‐to‐focus distance of five rings are 2.4 cm, 1.9 cm, and 1.8 cm for 4, 8, and 16 mm collimator, respectively. The fluence was created from the source film image with the mask filter. If the value of the pixel is greater than the threshold, the intensity is set to 1. The resulting fluence distribution was a map with inside pixels of intensity of 1 and the surrounding penumbra region with intensity between 0 and 1. The two regions change accordingly if the threshold changes. By iteratively adjusting these two parameters, optimal ring out factors were calculated to match the manufacturer data.

### D. Validation of the MC model: single‐shot plans and a clinical test case

The validation of the MC model was performed first by comparing calculated dose profiles of single‐shot plans with PFX TPS calculation for each collimator size. This served as the commissioning process of the model. Ring and collimator output factor and dose profiles were calculated with these simple setups. A clinical test case was used further to confirm the correctness of the implementation and to demonstrate the accuracy of the model. The clinical case was a clinical treatment plan for a skull base meningioma on the left side. The critical structure was the brain stem. The prescription was 12.5 Gy to the 50% isodose line. The plan was created with auto optimization, and it had a total of 9 shots with mixed collimators of all three collimator sizes. The conformality was 1.25. The dose algorithm was TMR 10. The MRI image was used for treatment planning. No CT image was taken. To compare with the LGP TPS calculation, we created a water phantom by exporting the skull geometry as a RT structure. A program read the structure and filled the voxels with water within the surface created by the structure since the algorithm TMR 10 in the LGP TPS assumes water material. The coordinates, collimator configurations, and irradiate time of the shots were used as an input to the MC simulation.

## III. RESULTS

### A. Relative dose profiles

**Figure 2:**
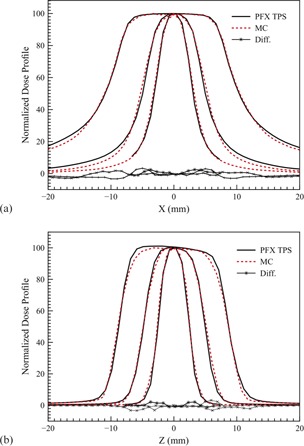
Relative dose profiles of 4, 8, and 16 mm collimators compared with PFX TPS along x‐axis (a) and z‐axis (b).

A digital spherical water phantom with the radius of 8 cm was used to calculate the dose distribution for the three collimators. The simulations were carried out on a high‐performance computing cluster with 40 nodes used in our calculations. The total simulated particles are $1\times 10^{9}$ and the calculation time was about 10 min for each run. The statistical uncertainty reached 0.4% for all calculations. Figs. 2(a) and (b) show the relative dose profiles (normalized to the central point) along x‐ and z‐axes for the three collimators, compared with PFX TPS calculations. We can see that very good agreement was achieved especially for the region of plateau (<2%). The larger difference comes from the penumbra region which is about 5%.

### B. Relative ring and collimator output factors

The center of the spherical phantom was set to position (0, 0, 0). To obtain the output factor, we use the voxel dose at the center which has a cubic volume of $1\;{\text{mm}}^{3}$ for the ratio calculation. The statistical uncertainty for this voxel is less than 0.5%. As discussed previously, we calculated the ring output factors for each collimator to match the values from the manufacturer by adjusting the source‐to‐focus distance and by fine‐tuning the source fluence map. The total collimator output factor can be derived directly from the summation of the ring output factors over five rings weighted by the number of sources at the ring for each collimator. As a different approach to verify the correctness of the used parameters, we performed MC calculations to obtain the total collimator output factor with a single shot. Table 1 lists the ring and total collimator output factors from our MC simulations, compared with the reference LGP values stored as default in the TPS by Elekta Instrument AB. As we can see, our collimator ROF results are within 0.7% of the manufacturer's values.

### C. Dose distribution comparison for the clinical test case

**Figure 3:**
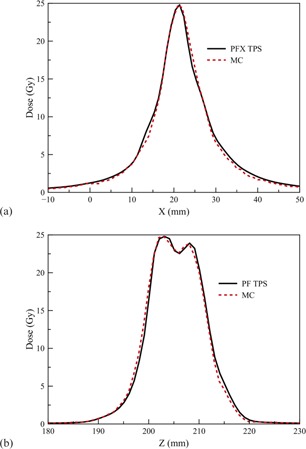
Dose profiles along x‐axis (a) and z‐axis (b) compared with PFX TPS for the clinical test case.

**Figure 4:**
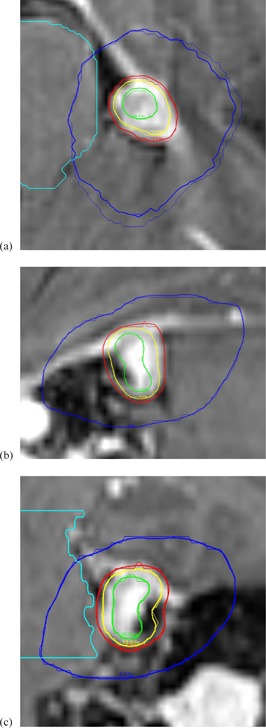
Illustration of MC calculated isodose lines overlay with PFX TPS for three planes: (a) axial, (b) sagittal, (c) cornal. MC: thick lines; TPS: thin lines. Blue: 3 Gy; red: 12.5 Gy; yellow: 15 Gy; green: 20 Gy.

**Figure 5:**
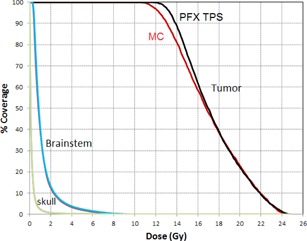
MC‐calculated DVHs for the target and organ at risk compared with PFX TPS for the clinical test case.

For the clinical test case, the MC‐calculated dose was converted into the RTDose format, and then was imported into a third‐party software (MIM Software, Cleveland, OH) for display and DVH calculation. The total simulated particles are $2\times 10^{9}$ on 20 processors. The calculation wall time was 10.3 min. The maximum statistical uncertainty for the region of 50% maximum dose was 0.62%. Figures 3(a) and (b) show the dose profile along X and Z direction, respectively, at the same point close to the maximum dose, compared with the LGP TPS calculation. Figures 4(a) to 4(c) show the dose contour comparison with the MRI image at three image planes. We can see the agreement is very well. The accumulative DVH is illustrated in Fig. 5. The DVHs for brainstem and for the whole skull are identical. For the tumor, the TPS plan gave 100% coverage of 50% isodose line (12.5 Gy), and the coverage calculated from the MC was 95.6%. The MC calculated D50, D70, D90, and D95 are 16.8 Gy, 14.9 Gy, 13.1 Gy, and 12.3 Gy, compared with 16.9 Gy, 15.2 Gy, 13.8 Gy, and 13.2 Gy from the LGP TPS calculation. The corresponding differences are ‐0.6%, ‐2.0%, ‐5.1%, and ‐6.8%.

## IV. DISCUSSION

The study of the air and bone tissue inhomogeneities in GK PFX radiosurgery treatment is of clinical relevance. Dose distribution for tumors close to the sinus and bones may vary greatly compared to the dose distribution calculated with water. A patient‐specific dose verification tool that is able to take inhomogeneity effect into account is clinically helpful. As the first step to achieve this goal, in this work, we developed a source model based on film measurement, and validated the model using simple shot configurations and a clinical case with generated homogenous phantom.

The MC simulation was implemented in such a way that the calculation time is not dependent on the number of shots. The location of the source is translated according to the shot coordinates so that the phantom stays still. The information of the source index was tagged to each bixel of the fluence map so that the correct transformation can be applied for difference shot coordinates. The sampling of the particle is taking from the overall fluence map, therefore it avoids calculating the dose shot‐by‐shot. We make use of 40 processors in a high‐performance computing cluster and generally simulate $1\times 10^{9}$ particles. The statistical uncertainty can reach less than 1% since the maximum radius of the tumor in GK surgery is usually less than 5 cm. The calculation wall time is about 10 min.

Approximations have been made in our source model. First, each source is assumed to be a single point source, although it is actually more like a line source. The slightly misalignment of the source and collimator axes for 8 and 16 mm collimators is ignored. Secondly, the electrons resulting from the photon collision with bushing assembly and stationary collimators are not taken into account. The scattered photons from collimation were taken into account only in terms of the energy, as the energy spectrum adopted in the work was generated from accurate *ab initio* approach.^(14)^ Thirdly, as we have no information of the detailed collimation system, we match the ring output factor with the manufacturer‐provided data by adjusting the source‐to‐focus distance of each ring and fine‐tuning the derived fluence map of each source. The processes were performed on a try‐and‐error basis. Hundreds of CPU times have been used to find the optimal results. To validate the MC model, we compared the simulated dose distributions of single‐shot and clinical plans with PFX TPS calculations. Good agreement from the comparisons justifies the approximations made in our MC model. Improvement of our model can utilize the phase space data at the cylinder surface generated from a detailed MC source simulation.

Future work will focus on studying the inhomogeneous effect in CT‐based voxel phantom and the difference from clinical treatment plans with PFX TPS algorithms. A framework that incorporates graphical user interface is currently under development. The beta version of the program will be available upon request in the near future.

## V. CONCLUSIONS

We have shown that a reasonable virtual source model can be created for LGP PFX MC simulations, based on film measurements. Overall, good agreement was achieved for dose profiles, especially for the region of plateau (<2%), while larger difference (<5%) came from the penumbra region when compared with the TPS. Good agreement was also obtained for a clinical treatment case. The DVHs for brainstem and the skull were almost identical and for the target the volume covered by the prescription (12.5 Gy to 50% isodose line) was 95.6% from MC calculation versus 100% from the TPS. This approach does not require the manufacturer's proprietary geometrical information, and we demonstrated that the accuracy of the model is adequate for a patient‐specific dose verification tool for the PFX treatments.

## ACKNOWLEDGMENTS

This work made use of the High Performance Computing Resource in the Core Facility for Advanced Research Computing at Case Reserve University.

## COPYRIGHT

This work is licensed under a Creative Commons Attribution 3.0 Unported License.

## Supporting information

Supplementary Material

Supplementary Material

Supplementary Material

Supplementary Material

Supplementary Material

Supplementary Material

Supplementary Material

Supplementary Material

Supplementary Material

Supplementary Material

Supplementary Material

Supplementary Material

Supplementary Material
