# Supplementary figures and images for: Development of a Monte Carlo model for treatment planning dose verification of the Leksell Gamma Knife Perfexion radiosurgery system
Source: J Appl Clin Med Phys. 2016 Jul 8;17(4):190–201. doi: 10.1120/jacmp.v17i4.6196 (PMC5690038; doi:10.1120/jacmp.v17i4.6196)

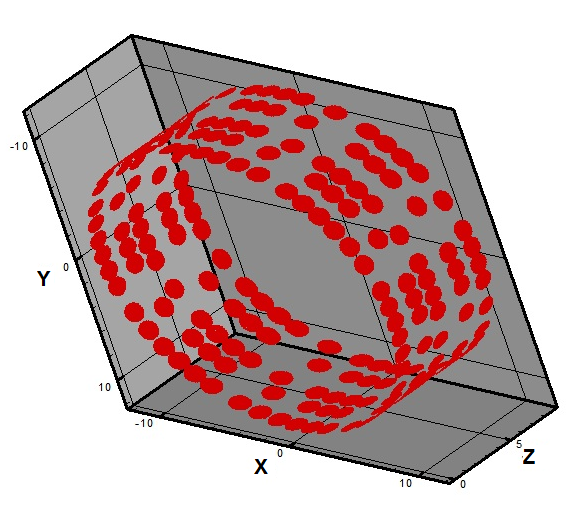

Supplement: Supplementary file 1 — Supplementary Material [file ACM2-17-190-s001.png]

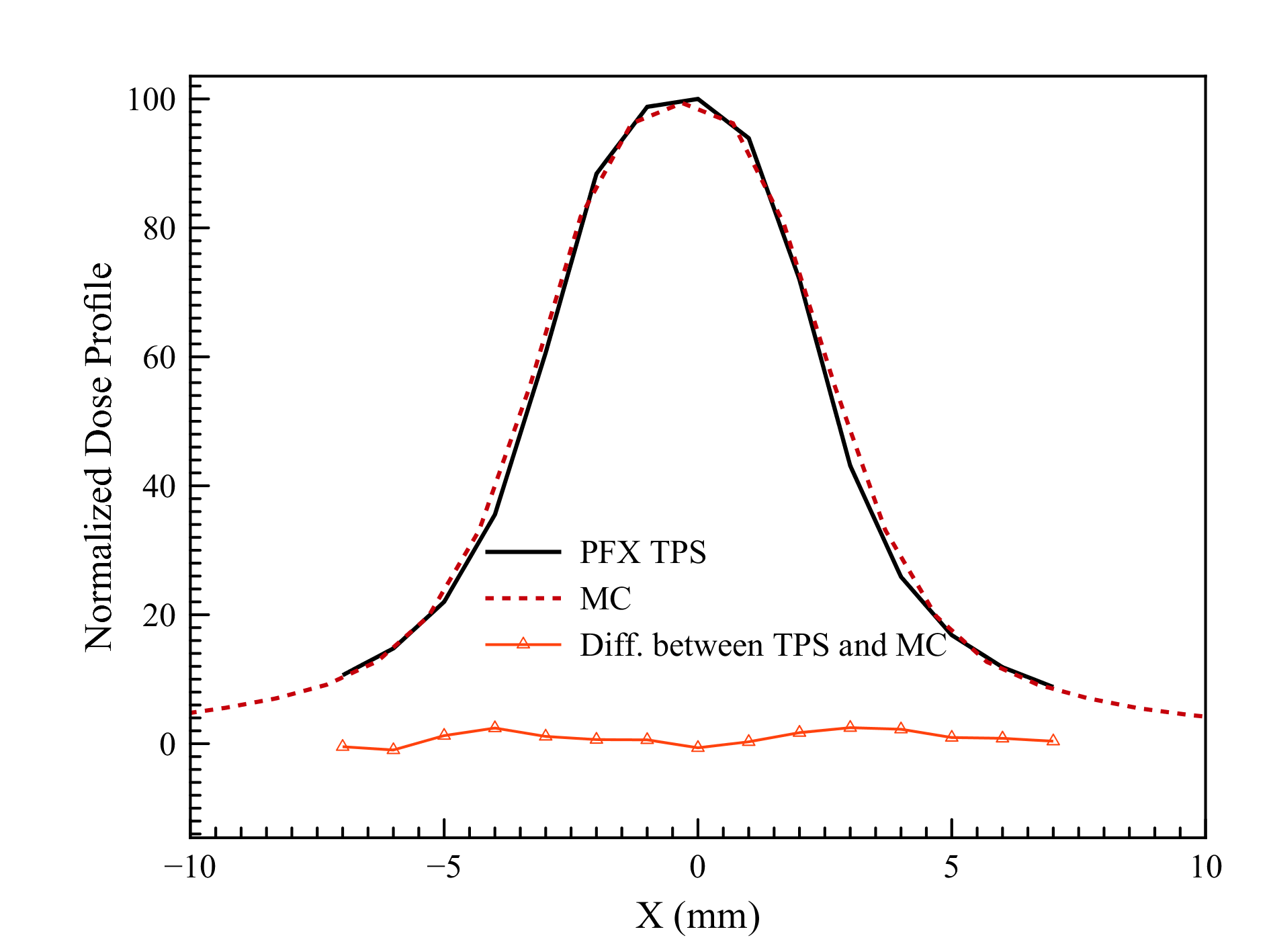

Supplement: Supplementary file 2 — Supplementary Material [file ACM2-17-190-s002.png]

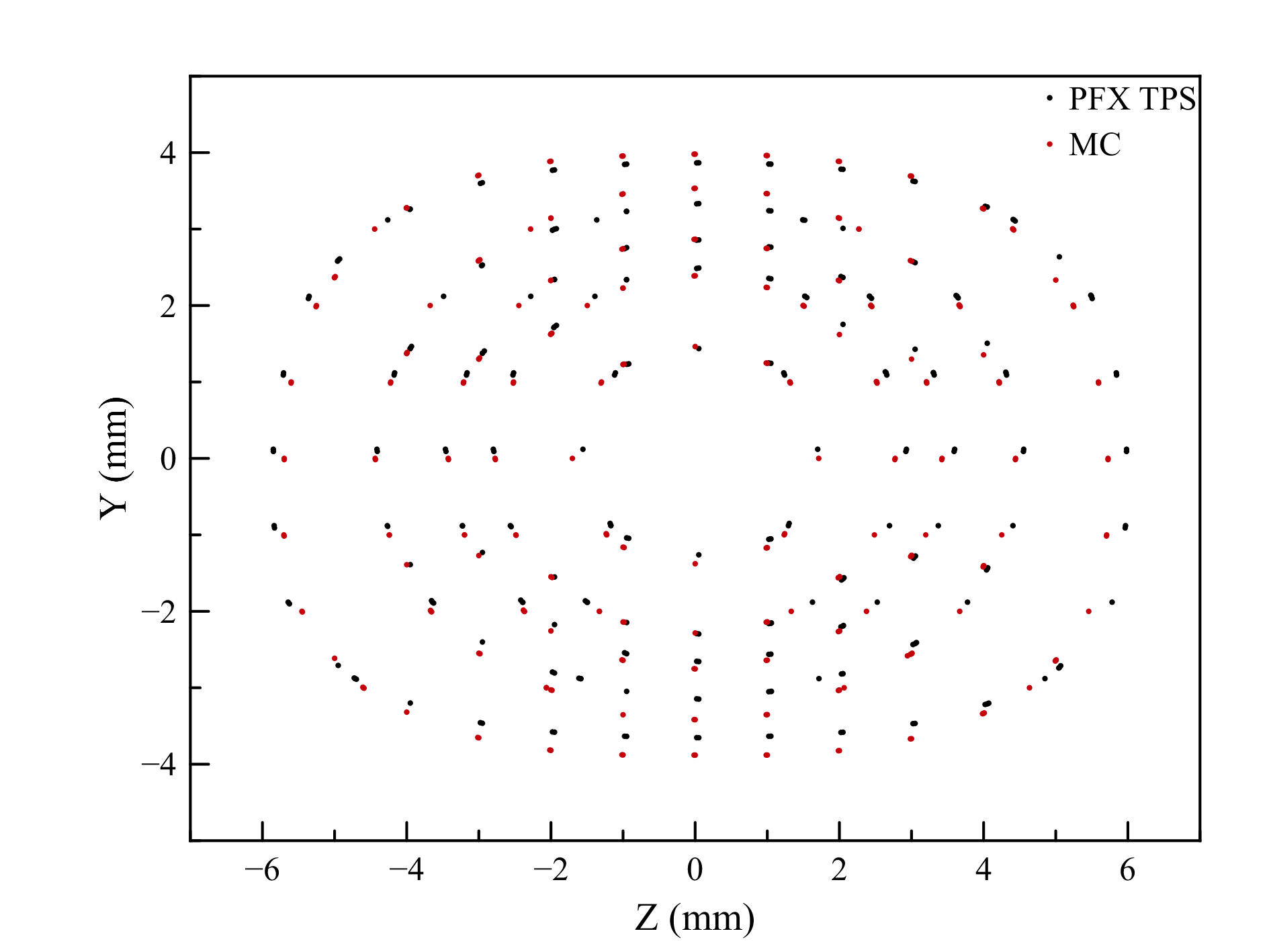

Supplement: Supplementary file 3 — Supplementary Material [file ACM2-17-190-s003.png]

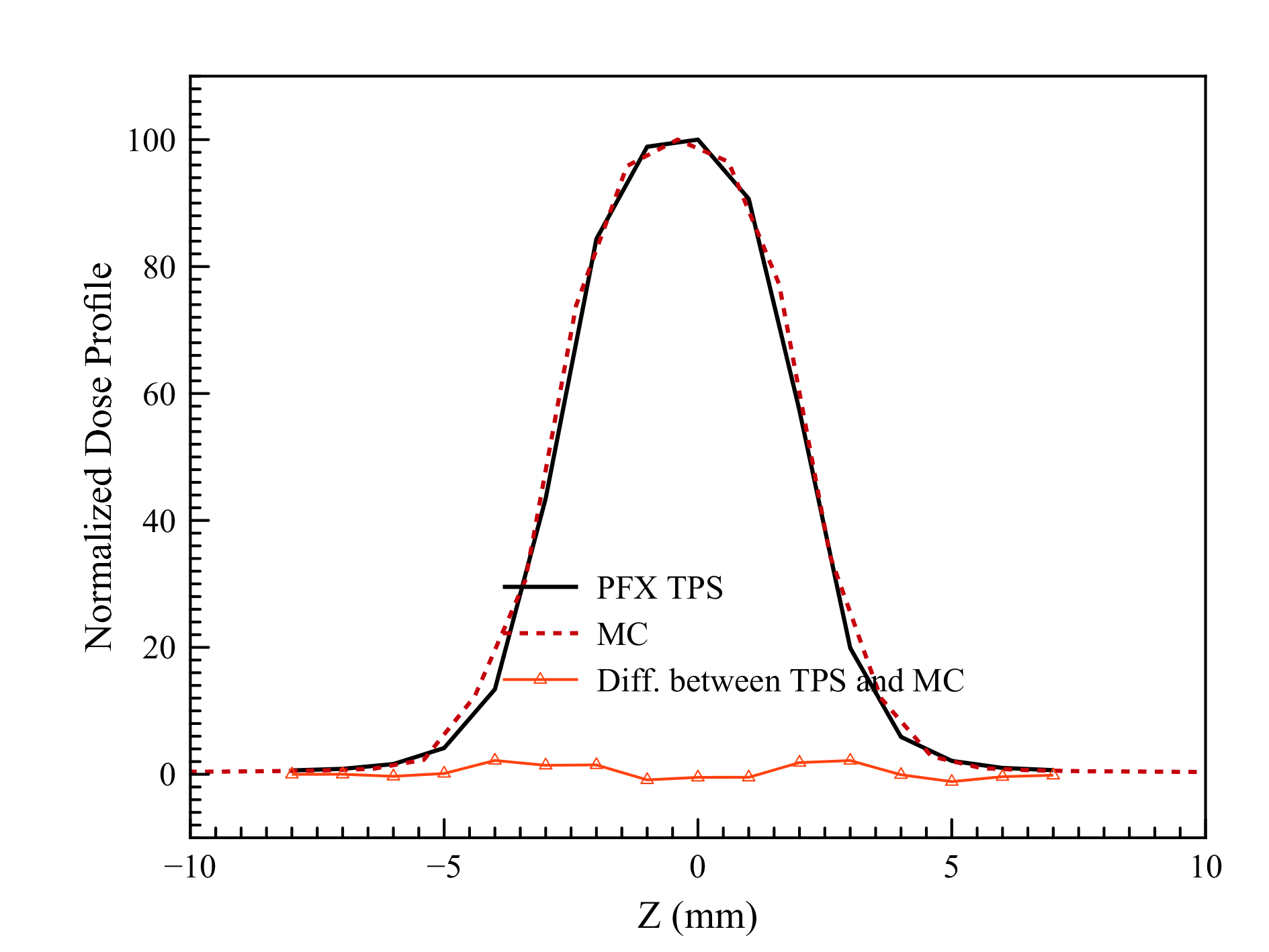

Supplement: Supplementary file 4 — Supplementary Material [file ACM2-17-190-s004.png]

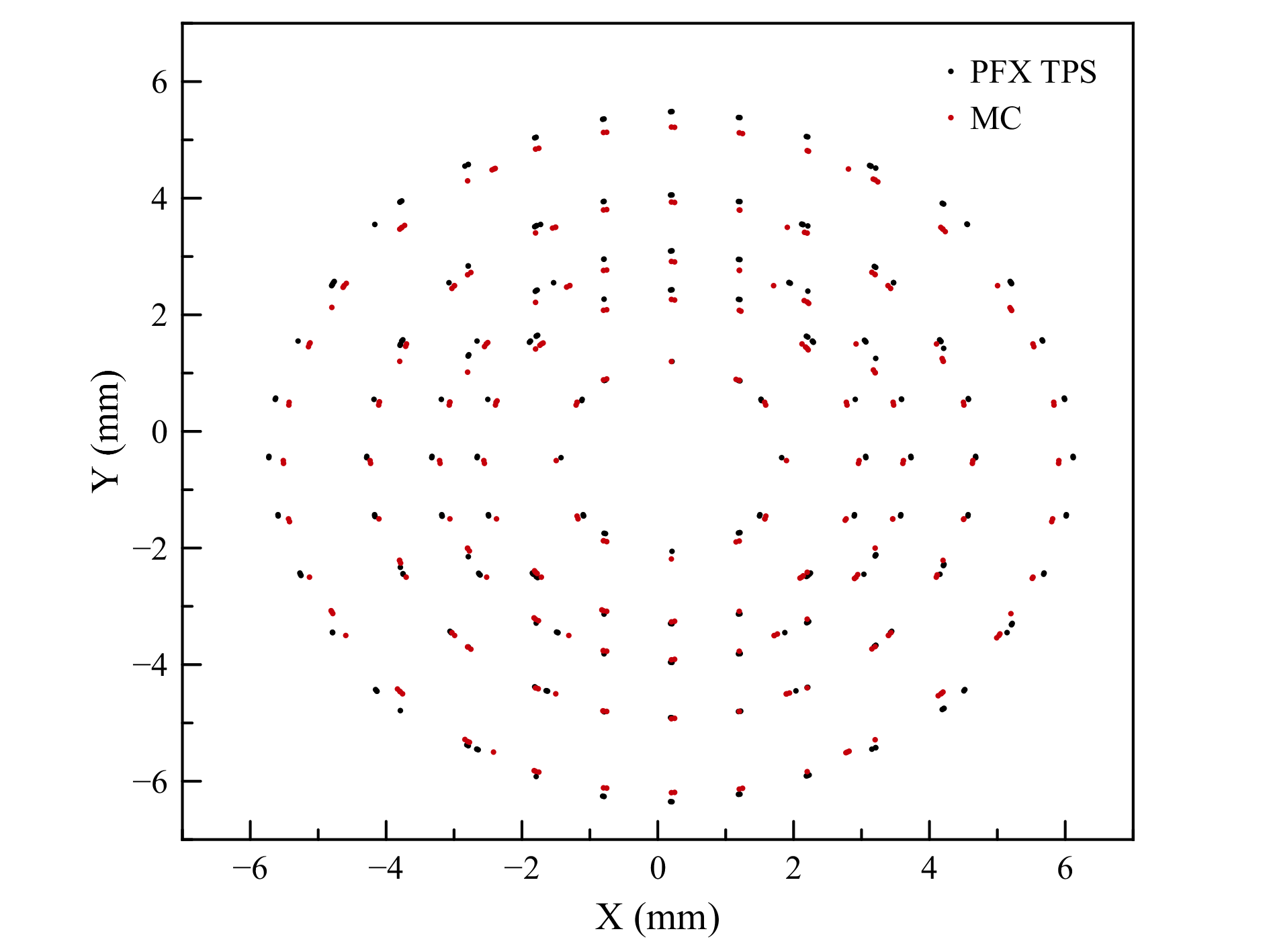

Supplement: Supplementary file 5 — Supplementary Material [file ACM2-17-190-s005.png]

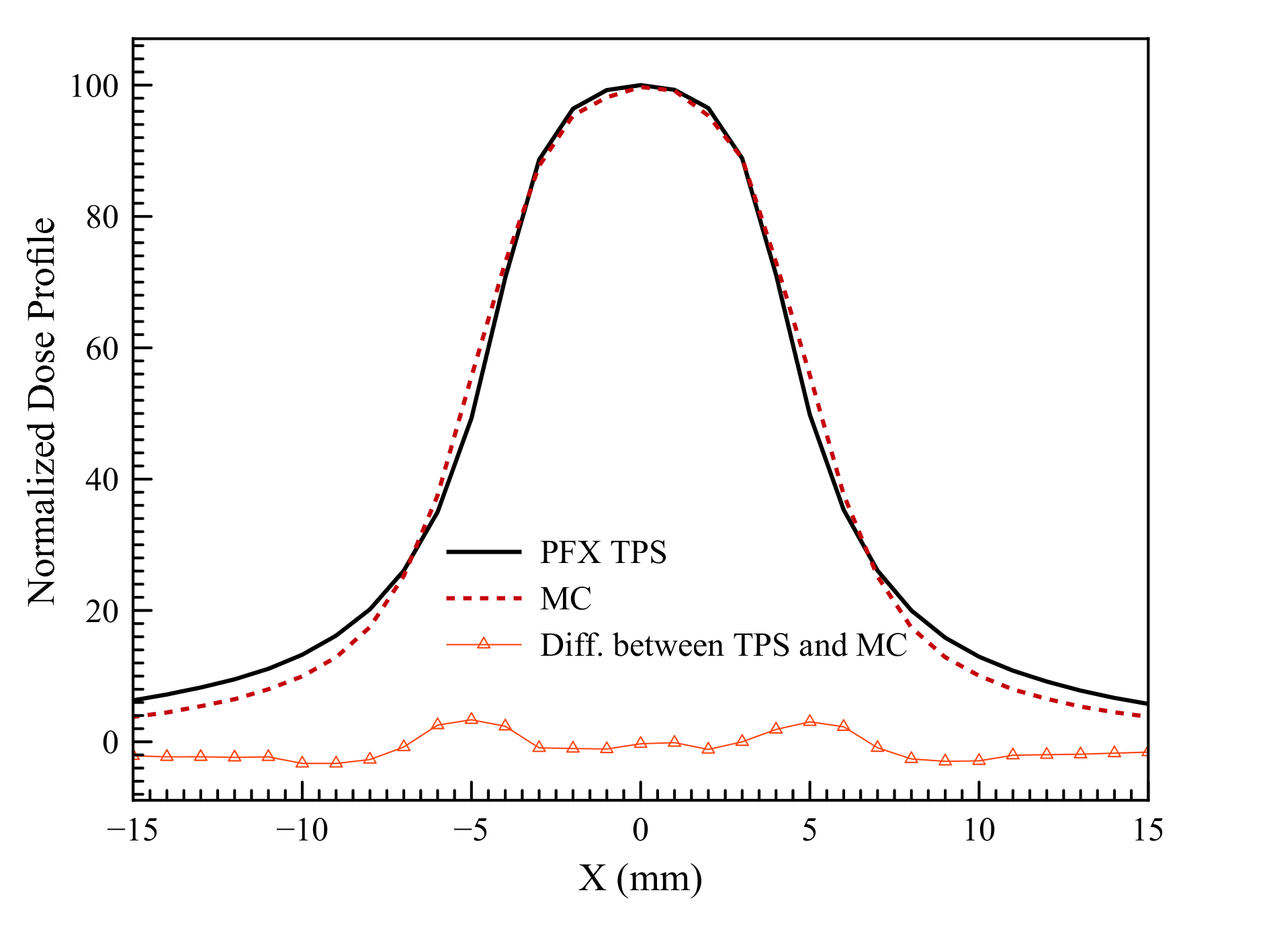

Supplement: Supplementary file 6 — Supplementary Material [file ACM2-17-190-s006.png]

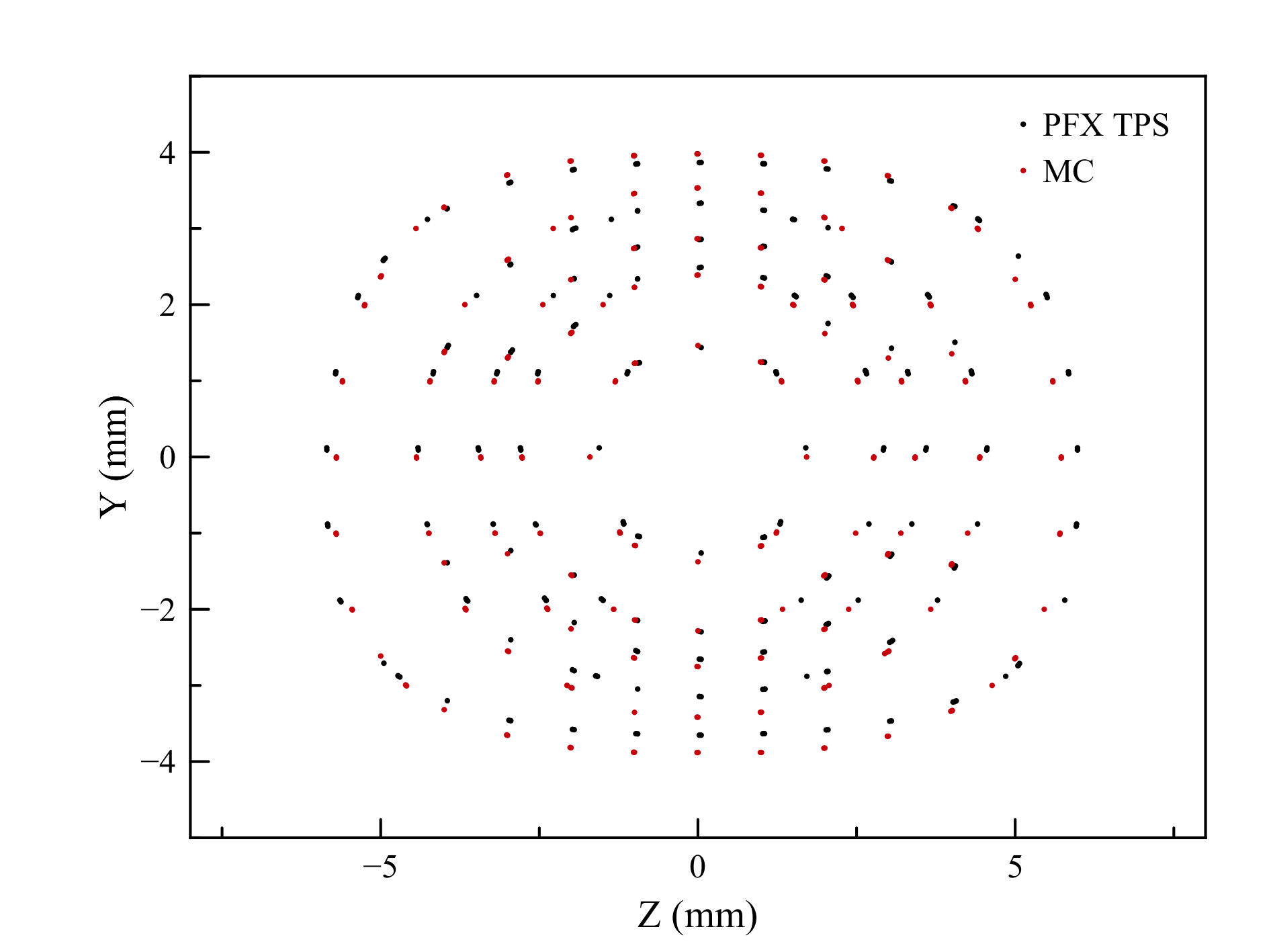

Supplement: Supplementary file 7 — Supplementary Material [file ACM2-17-190-s007.png]

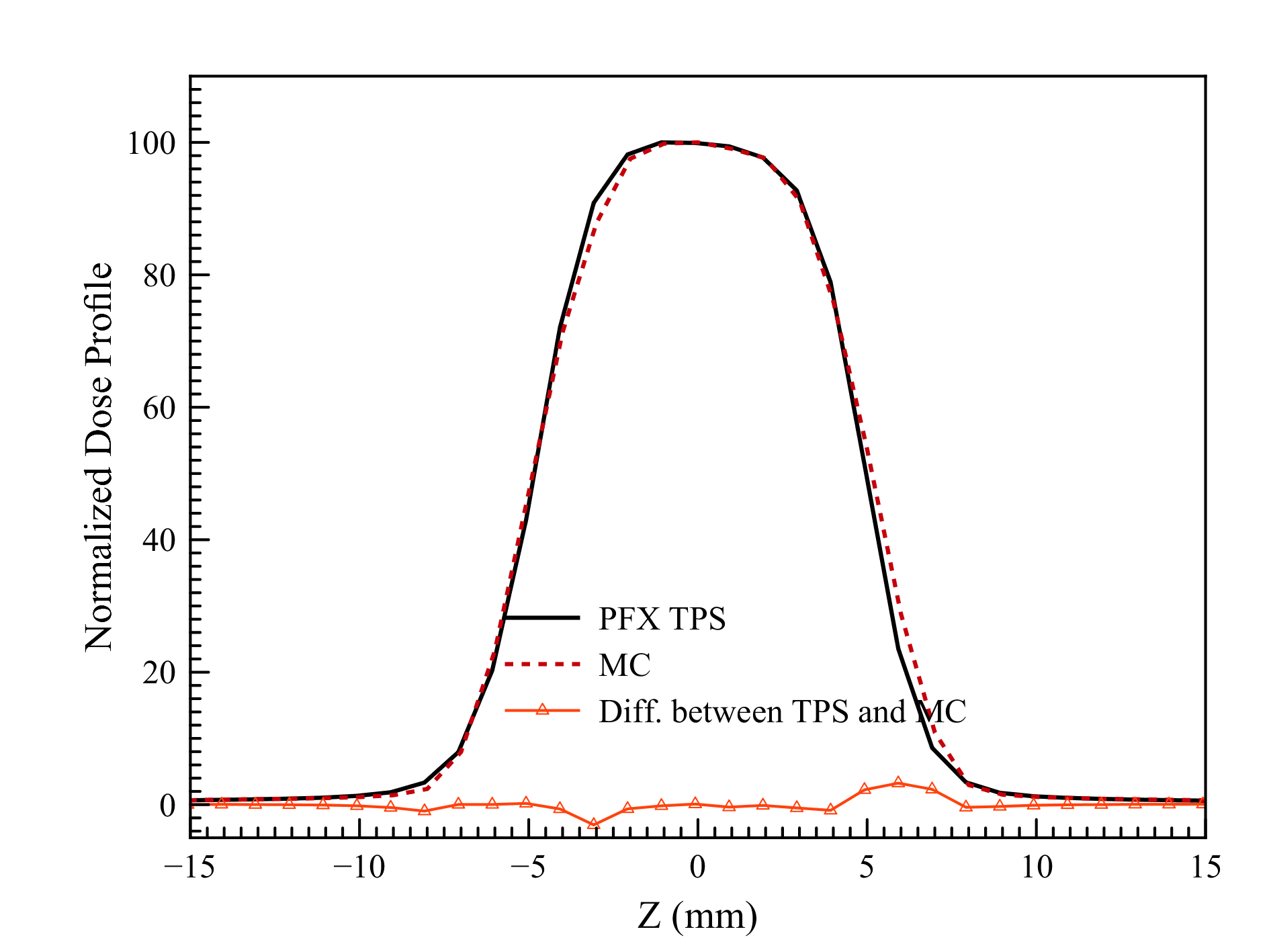

Supplement: Supplementary file 8 — Supplementary Material [file ACM2-17-190-s008.png]

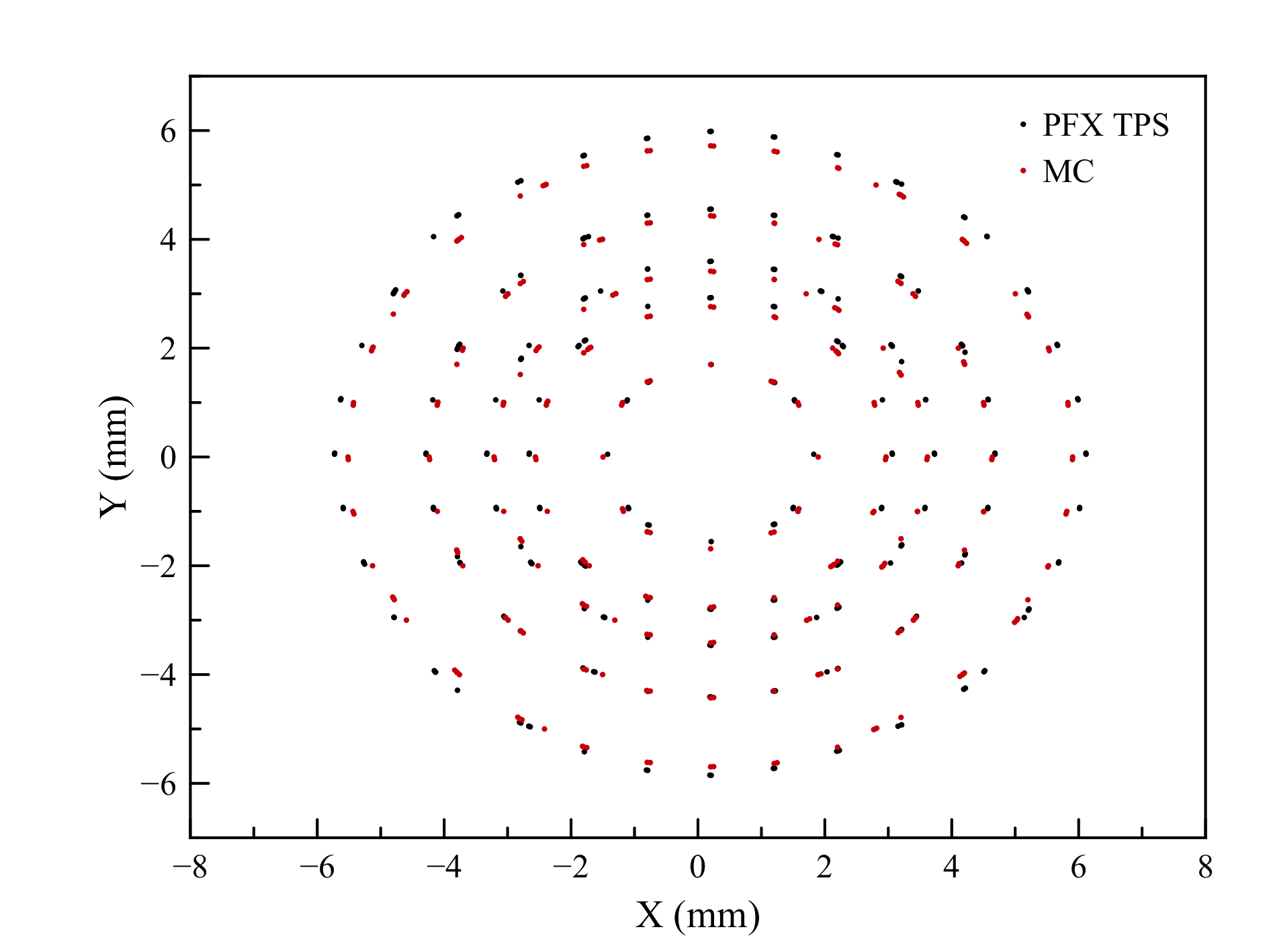

Supplement: Supplementary file 9 — Supplementary Material [file ACM2-17-190-s009.png]

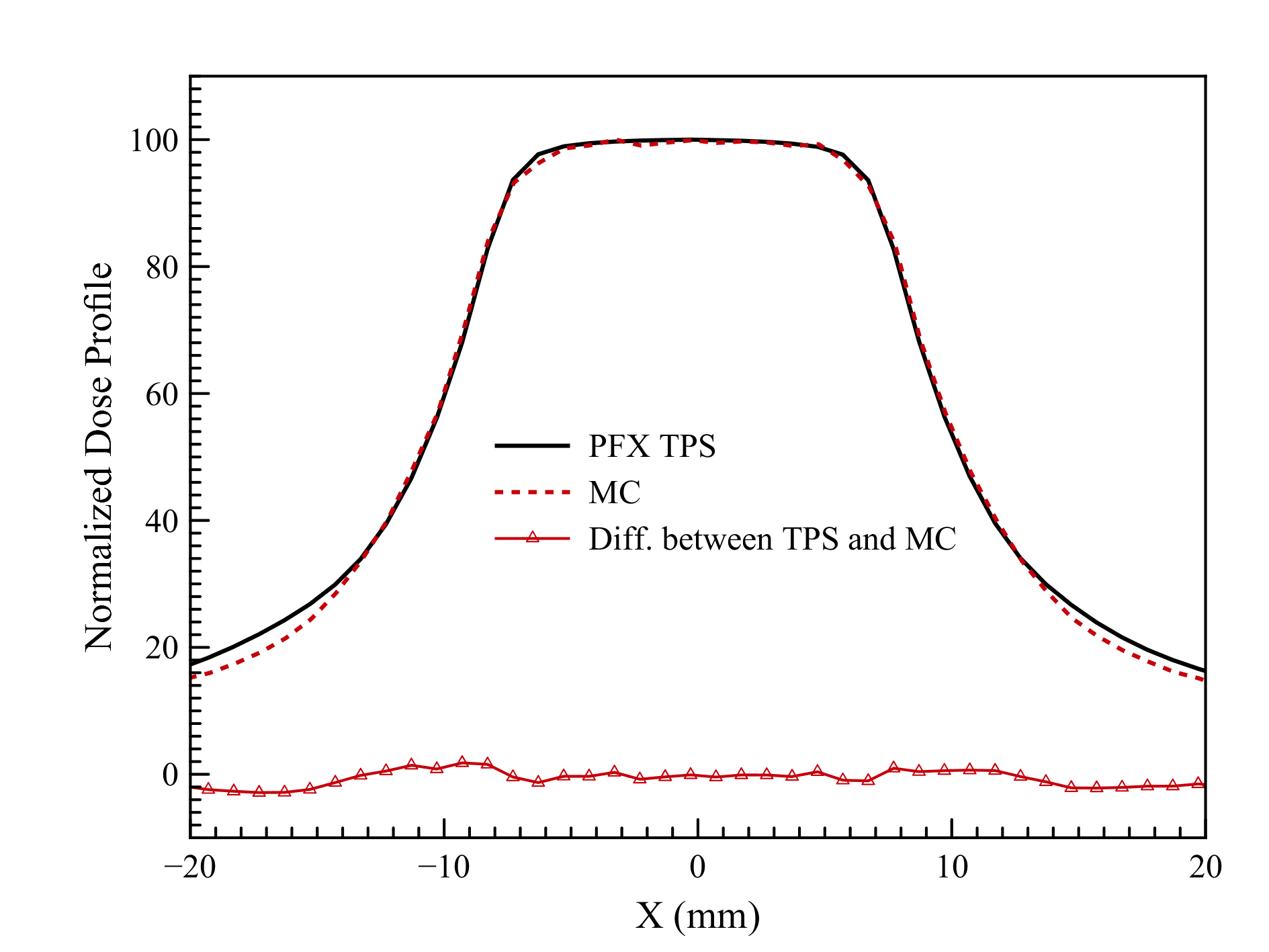

Supplement: Supplementary file 10 — Supplementary Material [file ACM2-17-190-s010.png]

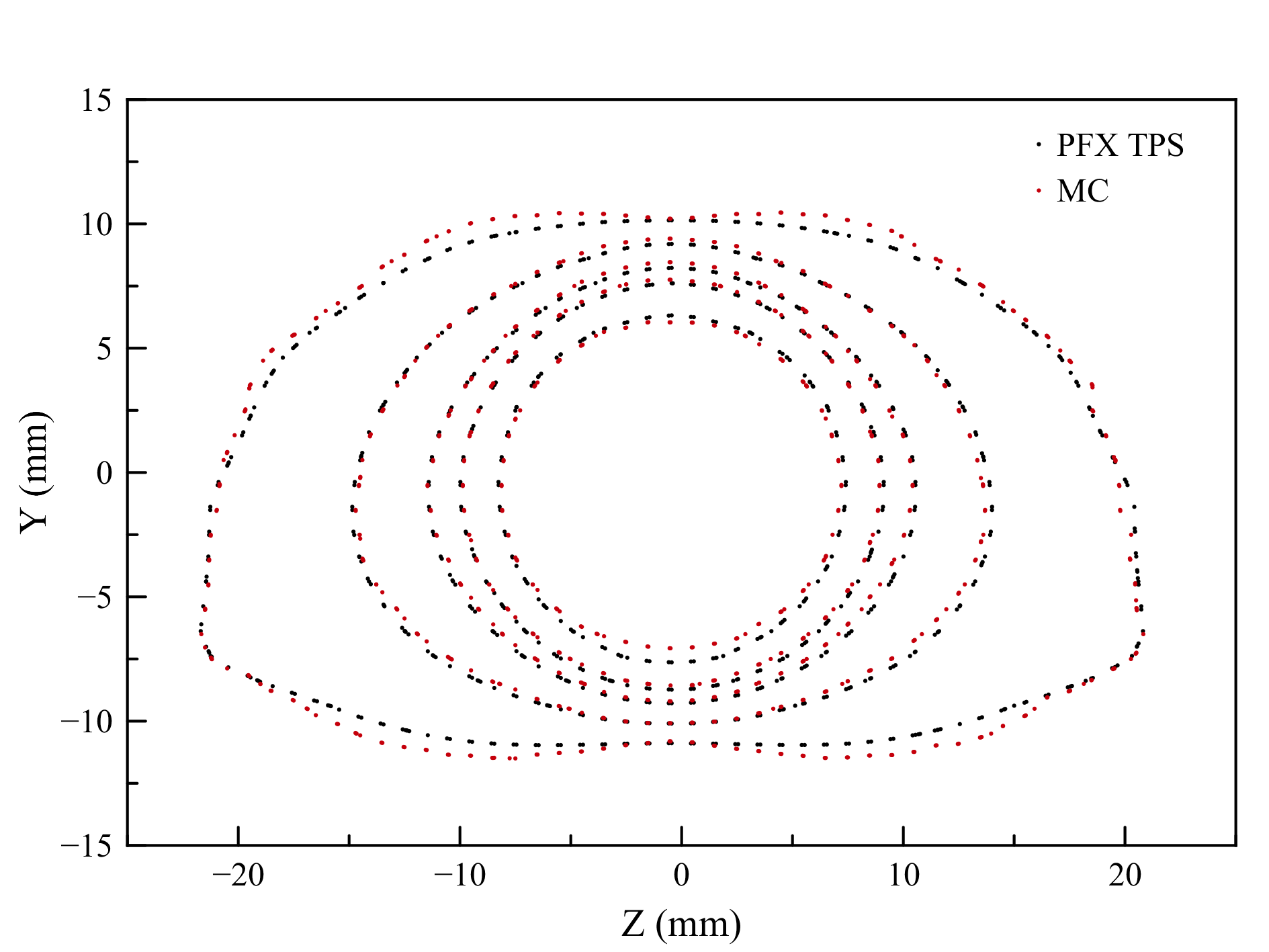

Supplement: Supplementary file 11 — Supplementary Material [file ACM2-17-190-s011.png]

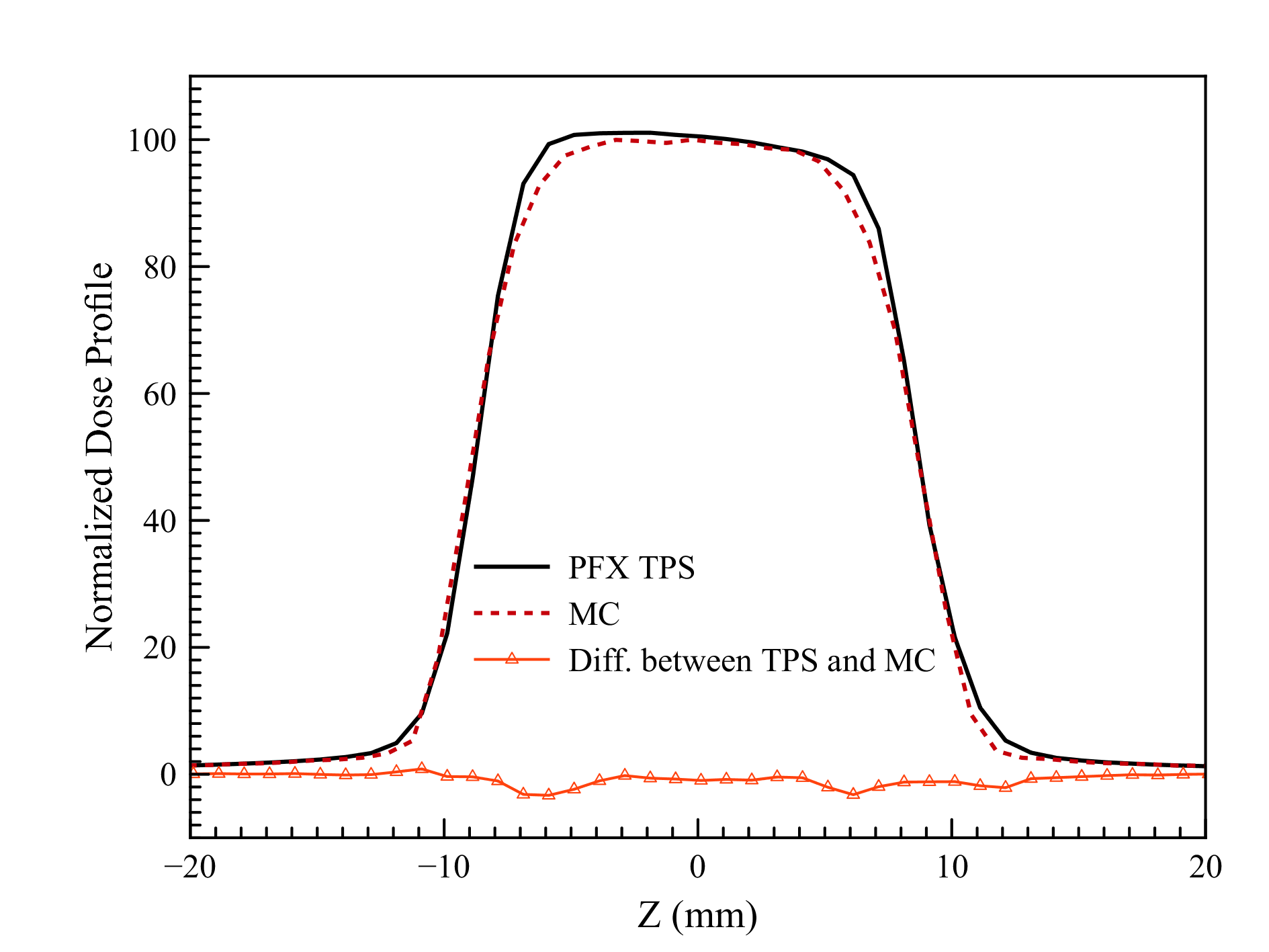

Supplement: Supplementary file 12 — Supplementary Material [file ACM2-17-190-s012.png]

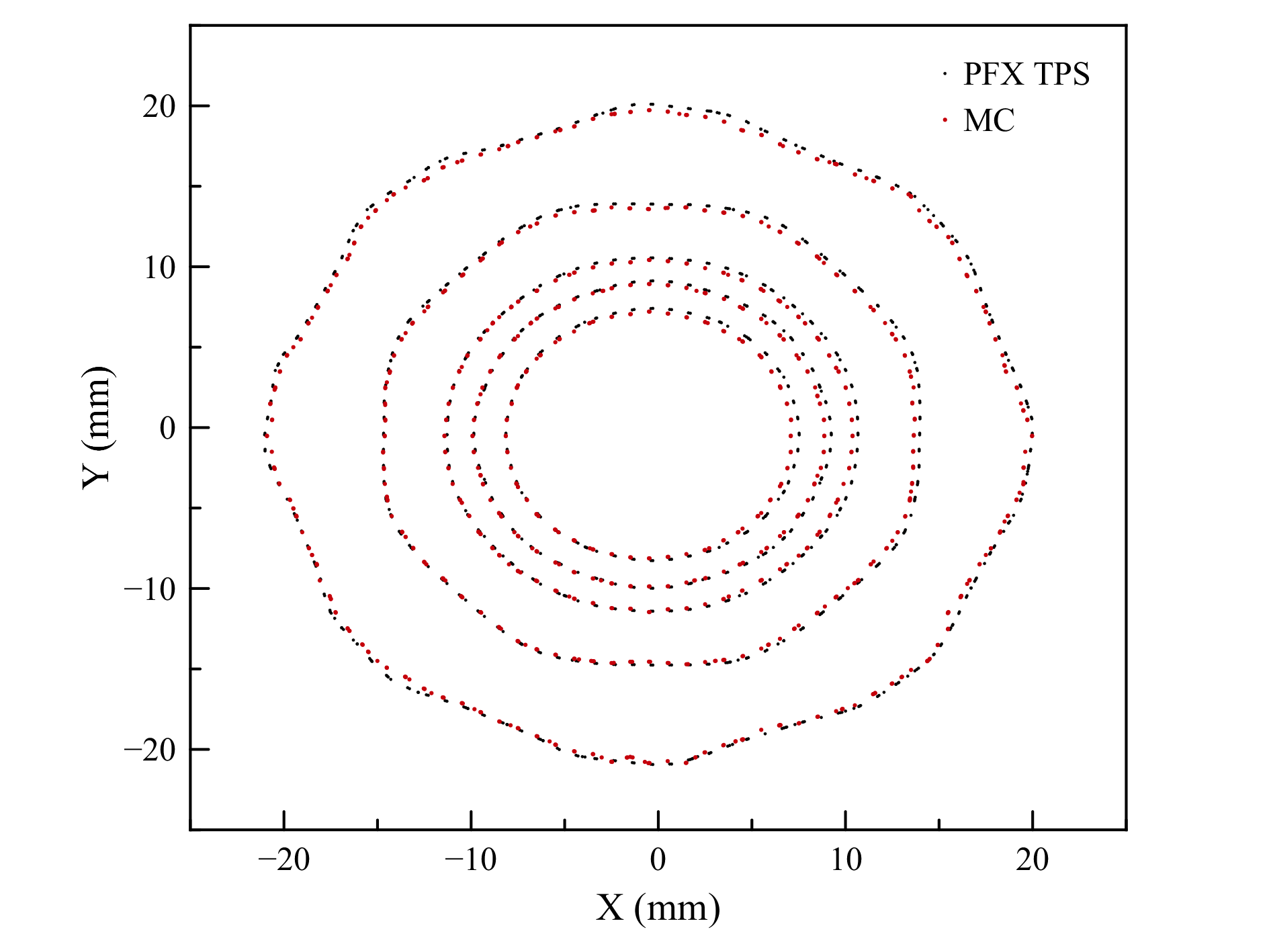

Supplement: Supplementary file 13 — Supplementary Material [file ACM2-17-190-s013.png]
